# Sex-related differences in myocardial fibrosis among patients with aortic stenosis: A systematic review and meta-analysis^☆^

**DOI:** 10.1016/j.ijcha.2025.101814

**Published:** 2025-09-30

**Authors:** Paul C. Onyeji, Shivank Dani, Sonise Momplaisir-Onyeji, Miguel C. Lenzi, Paweł Łajczak, Felipe S. Passos, Leo Consoli, Hristo Kirov, Torsten Doenst, Tulio Caldonazo

**Affiliations:** a All Saints University School of Medicine, Commonwealth of Dominica, Dominica; b GMERS Medical College and Hospital, Sola, Ahmedabad, India; c American University of Barbados, School of Medicine Faculty of Medicine, Barbados; d State University of Rio Grande do Norte (UERN), Brazil; e St. Jordana 18, 41-808 Zabrze, Medical University of Silesia, Poland; f Department of Thoracic Surgery, MaterDei Hospital, Salvador, Brazil; g Federal University of Bahia, School of Medicine, Salvador, Brazil; h Department of Cardiothoracic Surgery, Jena University Hospital, Jena, Germany; i Department of Cardiothoracic Surgery, Weil Cornell Medicine, NY, United States

**Keywords:** Myocardial fibrosis, Aortic stenosis, Late gadolinium enhancement, Extracellular volume, Septal E/e′

## Abstract

**Background:**

Aortic stenosis (AS) leads to pathological myocardial remodeling, particularly fibrosis, which contributes to adverse outcomes including heart failure, arrhythmias, and mortality. Evidence suggests sex-specific differences in fibrotic response, but individual studies are underpowered for definitive conclusions. This *meta*-analysis aimed to evaluate sex-related differences in myocardial fibrosis using cardiac magnetic resonance (CMR) parameters.

**Methods:**

Three databases were searched for studies comparing male and female patients with AS reporting CMR-derived measures. The primary outcomes were late gadolinium enhancement (LGE%), infarct-related and non-infarct-related LGE, extracellular volume (ECV) and Septal E/e′. Effect sizes were expressed as risk ratios (RR) for binary outcomes and mean differences (MD) for continuous outcomes, each with 95% confidence intervals (CI), using random-effects models. Study quality was appraised with the Newcastle–Ottawa Scale, and certainty of evidence was graded using the GRADE framework.

**Results:**

Seven studies (n = 2,105; 1,246 males) were included. No significant difference was observed in LGE% (MD 0.13; 95 %CI −0.93 to 1.18; p = 0.770), and risks of infarct-related LGE between sexes (RR 1.61; 95 %CI 0.90 to 2.89; p = 0.080). Males had higher risk of non-infarct LGE (RR 1.51; 95 %CI 1.34 to 1.70; p = 0.002). There were no significant differences in ECV (MD −0.45; 95 %CI −2.34 to 1.44; p = 0.506) and Septal E/e′ between sexes (MD −1.87; 95 %CI −4.05 to 0.32; p = 0.072).

**Conclusion:**

This *meta*-analysis shows sex-related differences in myocardial fibrosis in AS, with men exhibiting more focal replacement fibrosis and women a tendency toward diffuse interstitial fibrosis. These patterns highlight the relevance of incorporating sex-specific factors into diagnosis and management.

## 1 Introduction

Aortic stenosis (AS) is a prevalent valvular disease in which narrowing of the aortic valve imposes pressure overload on the left ventricle (LV). This condition triggers pathological myocardial remodeling, most notably myocardial fibrosis, characterized by excessive collagen deposition that stiffens the myocardium and impairs its function [[1], [2], [3]].

Emerging evidence highlights significant sex-based differences in this fibrotic response. Men with AS typically develop more pronounced LV hypertrophy and focal replacement fibrosis [4]. In contrast, women often have smaller cardiac dimensions and less replacement fibrosis, but may exhibit similar or greater diffuse fibrosis, as measured by extracellular volume (ECV) [5,6]. These variations, likely influenced by hormonal and genetic factors, are clinically significant, especially given the rising prevalence of AS in older populations [7,8].

This *meta*-analysis aims to systematically evaluate sex differences in myocardial fibrosis assessed by cardiac magnetic resonance (CMR). Particular attention is given to late gadolinium enhancement (LGE), ECV, and functional indices such as the septal E/e′ ratio, a well-established echocardiographic marker of diastolic dysfunction and adverse prognosis in cardiovascular disease [9,10].

## 2 Methods

The study selection followed the Preferred Reporting Items for Systematic Reviews and Meta-Analysis (PRISMA) statement guidelines [11,12]. The review was registered in the International Prospective Register of Systematic Reviews (PROSPERO ID CRD420251042301).

### 2.1 Search strategy

A comprehensive literature search was performed on MEDLINE, EMBASE, and Cochrane Library from inception to April 20, 2025, with the following search terms: “myocardial fibrosis”, “aortic stenosis”, “gender”, and “sex differences”. We also searched for additional studies using the references of previously included studies. The complete search strategy is available in Supplementary Table 1.

### 2.2 Study selection

Two independent reviewers (PO and SM) screened the records, after de-duplication. Any discrepancies and disagreements were resolved by a third author (TC). Titles and abstracts were reviewed against pre-defined inclusion and exclusion criteria.

### 2.3 Eligibility criteria

Inclusion in this *meta*-analysis was restricted to studies that met all the following criteria: (1) randomized controlled trials (RCT) or observational studies; (2) published in English; (3) directly comparing outcomes between male and female patients with myocardial fibrosis associated with aortic stenosis; and (4) reporting at least one predefined outcome of interest.

Exclusion criteria were: animal models, case reports, conference abstracts, reviews, and studies without a comparative design. For studies with unclear or overlapping populations, eligibility was resolved by consensus among reviewers, prioritizing the most comprehensive or recent dataset.

### 2.4 Quality assessment

The quality of included studies was assessed by two authors (ML and SD) using the Newcastle-Ottawa Scale (NOS) evaluation [13].

### 2.5 Data extraction and baseline characteristics

Two authors (PO and SM) independently performed data extraction using standardized forms that were piloted before full data collection. Any discrepancies were resolved by consensus, with arbitration from a third author when necessary. In cases where data was unavailable or ambiguous, this was explicitly reported and such studies were excluded from quantitative synthesis but retained for qualitative description when relevant. The extracted variables included study characteristics (author, year of publication, country, sample size, and reported outcomes) and patient demographics (age, sex, New York Heart Association [NYHA] class, diabetes mellitus [DM], and hypertension).

### 2.6 Outcomes

The primary outcomes were Late Gadolinium Enhancement (LGE), expressed as a percentage, infarct-related LGE, and non-infarct-related LGE. Secondary outcomes included extracellular volume (ECV) and diastolic function assessed by Septal E/e’.

### 2.7 Statistical analysis

Risk ratios (RR) with 95 % confidence intervals (CI) were calculated for binary outcomes, while Mean Difference (MD) was assessed for continuous outcomes. Heterogeneity was evaluated using Cochran’s Q test and quantified with the I2 statistic, which was interpreted as low (<25 %), moderate (25–50 %), or high (>50 %). A p-value < 0.05 was considered statistically significant for group differences. DerSimonian–Laird random-effects models were applied to all endpoints. In the presence of high heterogeneity, we planned a leave-one-out analysis. A subgroup analysis was also performed, excluding studies with a NOS score < 7, to evaluate the robustness of the findings. Publication bias was evaluated through visual inspection of the funnel plot, while Egger’s test was not performed due to the limited number of studies (<10). The Cochrane Handbook for Systematic Reviews of Interventions was followed for data handling and conversion. To ensure robustness of our findings, the certainty of evidence was assessed using the GRADE approach [14], incorporating five domains (risk of bias, inconsistency, indirectness, imprecision, and publication bias). All statistical analyses were performed using R (version 4.4.0, R Foundation for Statistical Computing, Vienna, Austria).

## 3 Results

### 3.1 Study characteristics

**Fig. 1:**
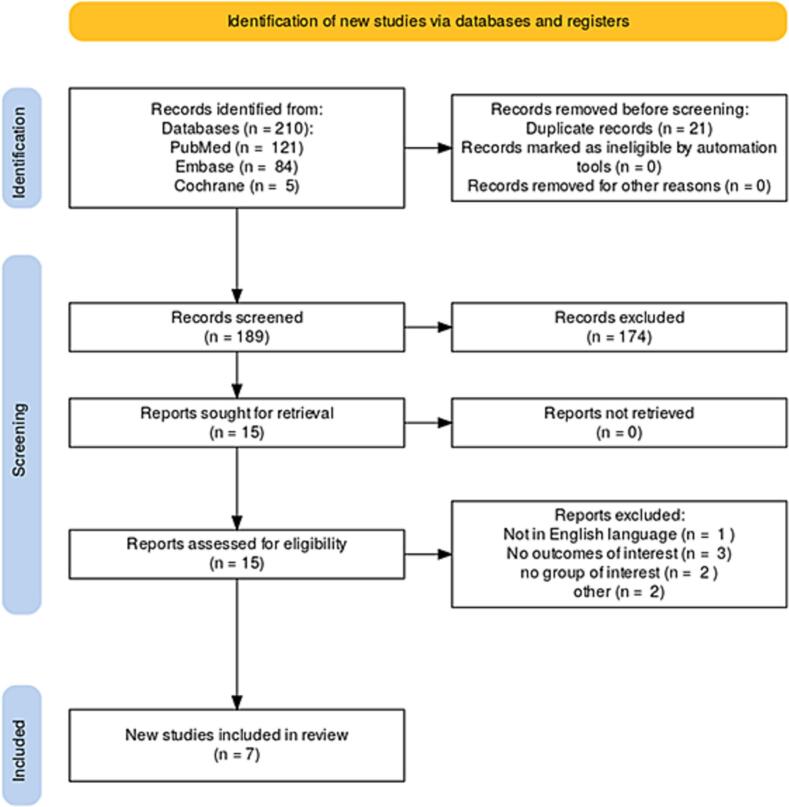
Preferred Reporting Items for Systematic Reviews and Meta-Analyses (PRISMA) flow diagram.

Fig. 1 shows the PRISMA flow diagram outlining the study selection process. The search strategy identified 210 results. After deduplication and exclusion based on title and abstract, 15 studies remained for full-text review. Of these, seven studies met all the inclusion criteria for the analysis [5,6,9,10,15,16,17].

### 3.2 Patient characteristics

The baseline characteristics of individual studies are presented in Table 1. Seven observational were included in this *meta*-analysis, encompassing 2,105 patients. Of these, 1,246 patients were male. The age ranged from 72 to 83 years. All cohorts demonstrated a male predominance, with the proportion of male participants ranging from 53 % to 70 %.

**Table 1:** Baseline characteristics of included studies.

Study	Country	Sample size, M/F (n)	Age Mean, y	Hypertension, M/F (n)	DM, M/F (n)	NYHA I, M/F (n)	NYHA II, M/F (n)	NYHA III, M/F (n)	NYHA IV, M/F (n)
Bhuva 2019	UK	63/53	70	79/71	21/19	NR	NR	NR	NR
Dobson 2016	UK	60/40	77	31/24	11/10	NR	NR	NR	NR
Kwak 2025	INT’L	368/302	72	178/161	80/66	99/53	129/113	92/93	9/12
Lee 2015	N/A	63/65	67	32/31	12/15	NR	NR	NR	NR
Singh 2019	UK	425/249	75	228/130	98/48	60/21	170/88	143/105	11/11
Tastet 2020	Canada	175/74	66	70/59	18/8	54/46	33/41	11/12	2/1
Treibel 2018	UK	92/76	70	81/73	22/29	23/10	40/39	26/28	4/1

DM: diabetes mellitus; F: Female; INT’L: international (Belgium, Canada, Germany, South Korea, UK, USA); M: male; NR: not reported; NYHA: New York Heart Association functional class; Y: years.

### 3.3 Quality assessment

The quality of the included studies was assessed using NOS evaluation (Supplementary Fig. 1). Three studies [5,6,15] achieved the highest score of 9/9, indicating outstanding methodological quality. Dobson et al. [16] received a score of 8/9, also classified as excellent. Tastet et al. [17] and Treibel et al. [10] and scored 6/9 and 7/9, respectively, reflecting moderate to high and moderate quality. Lee et al. [9] received a score of 5/9, suggesting potential methodological limitations.

### 3.4 Primary outcomes

**Fig. 2:**
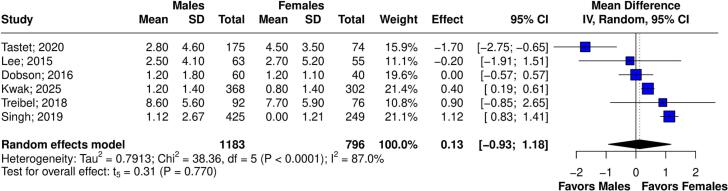
Forest plots comparing LGE% in male and female patients.

**Fig. 3:**
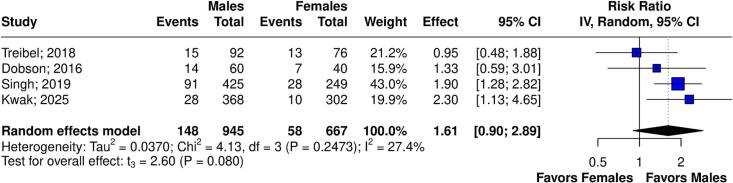
Forest plots comparing infarct-related LGE in male and female patients.

**Fig. 4:**
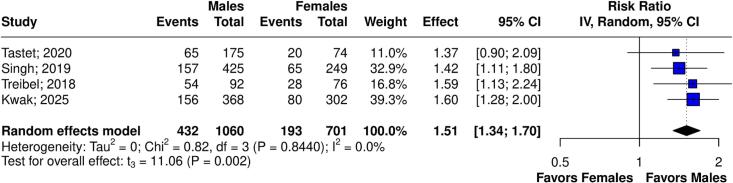
**.** Forest plots comparing non-infarct LGE in male and female patients.

Table 2 summarizes the *meta*-analysis findings. There was no significant difference in LGE% between sexes (6 studies, 1,979 patients; MD 0.13; 95 % CI −0.93 to 1.18; p = 0.770, I^2^ = 87 %; Fig. 2), and infarct-related LGE (4 studies, 1,612 patients; RR 1.61; 95 % CI 0.90 to 2.89; p = 0.080, I^2^ = 27 %; Fig. 3). Males had a higher risk of non-infarct LGE (4 studies, 1,761 patients; RR 1.51; 95 % CI 1.34 to 1.70; p = 0.002, I^2^ = 0 %; Fig. 4). The visual inspection of funnel plot reveals no significant asymmetry (Supplementary Fig. 2A-C).

**Table 2:** Summary of outcomes.

Outcome	Number of Studies	Number of Patients	Effect Estimate, Random Model (95 % CI, p-value)
LGE% of Myocardium	6	1979	(MD 0.13; 95 % CI −0.93 to 1.18; p = 0.770, I^2^ = 87 %)
Infarct-related LGE	4	1612	(RR 1.61; 95 % CI 0.90 to 2.89; p = 0.080, I^2^ = 27 %)
Non-infarct-related LGE	4	1761	(RR 1.51; 95 % CI 1.34 to 1.70; p = 0.002, I^2^ = 0 %)
Extracellular volume	4	1203	(MD −0.45; 95 % CI −2.34 to 1.44; p = 0.506, I^2^ = 88 %)
Septal E/e’	4	1207	(MD −1.87; 95 % CI −4.05 to 0.32; p = 0.072, I^2^ = 59 %)

CI: confidence interval; MD: mean difference; RR: relative risk; LGE: late gadolinium enhancement.

### 3.5 Secondary outcomes

**Fig. 5:**
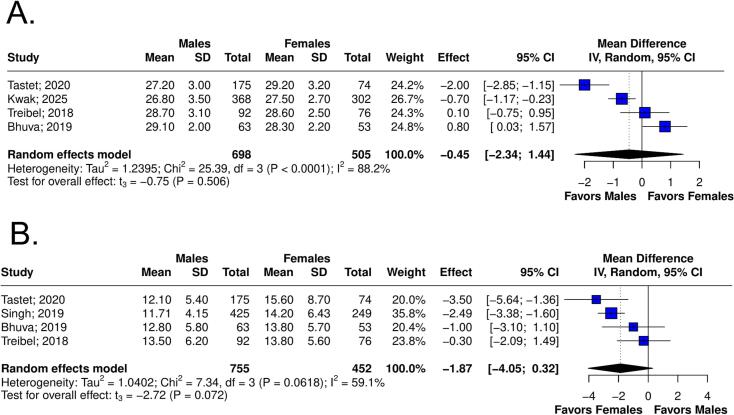
**.** Forest plots comparing outcomes in male and female patients. (A) Extracellular volume. (B) Septal E/e’ ratio.

Males showed no significant difference in ECV compared to females (4 studies, 1,203 patients; MD −0.45; 95 % CI −2.34 to 1.44; p = 0.506, I2 = 88 %; Fig. 5A). Septal E/e’ was significantly lower in males (4 studies, 1,207 patients; MD −1.87; 95 % CI −4.05 to 0.32; p = 0.072, I2 = 59 %; Fig. 5B).

### 3.6 Sensitivity analysis

To address the robustness of the findings, a leave-one-out sensitivity analysis was performed for all outcomes that demonstrated high heterogeneity. For LGE%, the results remained non-significant when individual studies were omitted. A similar pattern was observed for ECV. In contrast, for septal E/e′, statistical significance was reached when the study by Treibel et al. [10] was excluded (Supplementary Figs. 3–5). Additionally, a subgroup analysis stratifying studies by NOS score was conducted for all outcomes, which revealed no meaningful changes in the pooled estimates (Supplementary Figs. 6–10).

### 3.7 GRADE certainty of proof

Supplementary Table 2 summarizes GRADE overview of the results. Due to limits in study design (observational), moderate consistency, imprecision (large confidence intervals), and possible risk of bias, the certainty of evidence ranged from very low to low across outcomes. No major issues with publication bias or indirectness surfaced.

## 4 Discussion

In this systematic review and *meta*-analysis of seven studies including 2105 patients, we comprehensively analyzed sex differences in myocardial fibrosis secondary to aortic stenosis. Our main findings were as follows: (I) there was no significant difference in LGE% of myocardium between sexes; (II) males had a higher risk of non-infarct-related LGE, whereas infarct-related LGE showed only a nonsignificant trend; (III) extracellular volume did not differ significantly between sexes; and (IV) there was no significant difference in septal E/e′ between sexes.

Histopathological studies have consistently demonstrated that myocardial fibrosis is commonly related to AS and is strongly associated with increased morbidity and mortality [7]. Myocardial fibrosis, characterized by excessive collagen deposition and stiffening of the myocardium, leads to impaired ventricular compliance and function. Diffuse interstitial fibrosis, quantified by ECV, may exhibit some reversibility after aortic valve replacement (AVR). In contrast, focal replacement fibrosis, detected as LGE on cardiac imaging, is generally considered irreversible [18]. In addition, histological studies have shown higher collagen content and greater expression of profibrotic genes in male myocardium compared to females, supporting imaging findings of increased focal fibrosis in men [6,10].

Our analysis revealed sex-specific patterns in myocardial remodeling among patients with severe symptomatic AS. In male patients, myocardial fibrosis appears to be predominantly influenced by the severity of AS and the degree of LV hypertrophy. Studies such as Dobson et al. [16] and Treibel et al. [10] reported higher levels of LGE in men, including non-infarct patterns, suggesting a greater burden of replacement fibrosis. Conversely, Tastet et al. [17] highlighted that women, despite having smaller LV mass and sizes, exhibited higher levels of diffuse fibrosis as measured by ECV, regardless of AS severity. However, other large multicenter cohorts have reported similar ECV% between sexes, possibly reflecting differences in study populations, imaging protocols, or disease stage at assessment [6,17]. This suggests that diffuse fibrosis in women may involve mechanisms beyond hemodynamic stress alone.

Importantly, while men tend to develop more pronounced hypertrophy and larger LV dimensions—often accompanied by a history of coronary artery disease (CAD) and higher prevalence of focal fibrosis—women with severe AS are typically older and exhibit smaller hearts with less concentric hypertrophy. These differences suggest sex-specific adaptive responses to pressure overload. Kwak et al. [6] found that women generally demonstrated less wall thickening, hypertrophy, LGE, and replacement fibrosis compared to men, although ECV% was similar across sexes. This aligns with the notion that women may predominantly accumulate diffuse interstitial fibrosis, whereas men are more prone to replacement fibrosis.

The biological mechanisms underlying these differences remain incompletely understood but likely involve hormonal influences and sex-specific gene expression. Transcriptomic and immunohistochemical analyses indicate that male myocardium under pressure overload upregulates profibrotic and inflammatory pathways, while female myocardium shows relative repression of these pathways. The ovarian hormone 17β-estradiol (E2) has been shown in experimental models to suppress collagen synthesis in female cardiac fibroblasts, whereas testosterone appears to promote maladaptive remodeling, exacerbating hypertrophy and fibrosis in male hearts [19,20].

Current evidence further supports that estrogen confers antifibrotic effects and promotes favorable remodeling, while testosterone is linked to increased fibrosis and adverse remodeling [19]. These hormonal influences also help explain the paradox of similar ECV but different LGE patterns: ECV quantifies diffuse interstitial fibrosis, which may be comparable across sexes, whereas LGE captures focal replacement fibrosis, more prevalent in men. Accordingly, women tend to accumulate diffuse interstitial fibrosis, whereas men are predisposed to focal replacement fibrosis, reflecting distinct molecular and cellular remodeling pathways. [21].

The clinical implications of these findings are significant. Diffuse fibrosis, even if partially reversible after AVR, may contribute to the higher incidence of heart failure with preserved ejection fraction (HFpEF) observed in women. On the other hand, replacement fibrosis in men is a well-recognized predictor of adverse outcomes, including arrhythmias and sudden cardiac death. Notably, women may experience more rapid regression of LV hypertrophy and fibrosis following AVR, potentially due to their lower baseline burden of replacement fibrosis and more favorable molecular remodeling profiles [6,19].

Lee et al. [9] reported no significant difference in LGE extent between sexes but underscored its association with poor prognosis in both genders, particularly among men [13].

The included studies employed ECV thresholds ranging from 27 % to 30 %, reflecting slight variations due to differences in imaging protocols and reference populations. These thresholds represent myocardial extracellular matrix expansion beyond normal ranges and correlate with functional impairment. Diagnostic criteria for severe AS were consistent across studies, adhering to echocardiographic guidelines: aortic valve area ≤1.0 cm^2^, mean transvalvular gradient ≥40 mmHg, or peak aortic jet velocity ≥4.0 m/s.

Overall, our synthesis indicates that men are more prone to replacement fibrosis, whereas women more frequently develop diffuse interstitial fibrosis, patterns that carry distinct prognostic and therapeutic implications. The greater burden of replacement fibrosis in men, detected by LGE, may justify earlier intervention in selected patients to prevent the progression of irreversible myocardial damage and its arrhythmic consequences. Conversely, the predominance of diffuse fibrosis in women, partially reversible after AVR, underscores the potential for favorable remodeling even when surgery is performed at more advanced stages of disease. This supports the incorporation of sex-specific factors into decisions regarding the optimal timing of AVR and patient selection. Furthermore, future risk stratification algorithms could integrate sex as a modifier variable, combined with CMR-derived markers of fibrosis, to refine prognosis and guide tailored management strategies.

While our analysis focused on CMR-derived parameters, it is important to acknowledge the growing role of alternative imaging modalities for fibrosis assessment in patients with aortic stenosis. Cardiac computed tomography (CCT) deserves attention, as it is routinely performed as part of the pre-interventional evaluation in candidates for aortic valve replacement. Recent evidence, including a comprehensive *meta*-analysis [22], demonstrated that patients with elevated CT-ECV values had a significantly worse medium-term prognosis following valve replacement compared with those with normal CT-ECV values. Incorporating CT-based fibrosis assessment alongside CMR may therefore enhance risk stratification and improve clinical decision-making in this population.

### 4.1 Study strengths and limitations

This is the first *meta*-analysis to evaluate gender-related differences in myocardial fibrosis secondary to AS, addressing multiple outcomes including infarct and non-infarct LGE. While the low heterogeneity for infarct and non-infarct LGE supports the robustness of these findings, limitations inherent to study-level *meta*-analyses remain. Methodological heterogeneity, residual confounding, and lack of patient-level data restricted more detailed subgroup analyses, particularly according to AVR status, aortic stenosis severity, and the presence of coronary artery disease. Variability in imaging protocols, definitions of fibrosis thresholds, and the disproportionate weight of larger studies (Singh et al., Kwak et al.) may also have influenced results. The higher heterogeneity observed in septal E/e’, LGE percentage, and ECV% likely reflects differences in study populations and measurement techniques. Further prospective studies with standardized CMR protocols are warranted to clarify the clinical significance of these sex-specific differences and their implications for tailored management strategies.

## 5 Conclusion

This *meta*-analysis shows sex-related differences in myocardial fibrosis in AS, with men exhibiting more focal replacement fibrosis and women a tendency toward diffuse interstitial fibrosis. These patterns highlight the relevance of incorporating sex-specific factors into diagnosis and management.

## CRediT authorship contribution statement

**Paul C. Onyeji:** Writing – original draft, Methodology, Investigation, Formal analysis, Conceptualization. **Shivank Dani:** Writing – original draft, Methodology, Investigation, Formal analysis. **Sonise Momplaisir-Onyeji:** Writing – original draft, Software, Data curation. **Miguel C. Lenzi:** Writing – original draft, Methodology, Investigation, Conceptualization. **Paweł Łajczak:** Methodology, Investigation, Data curation. **Felipe S. Passos:** Writing – review & editing, Methodology. **Leo Consoli:** Software, Resources, Methodology. **Hristo Kirov:** Writing – review & editing, Project administration. **Torsten Doenst:** Writing – review & editing. **Tulio Caldonazo:** Writing – review & editing, Validation, Funding acquisition.

## Funding

Tulio Caldonazo was funded by the Deutsche Forschungsgemeinschaft (DFG, German Research Foundation) Clinician Scientist Program OrganAge funding number 413668513, by the Deutsche Herzstiftung (DHS, German Heart Foundation) funding number S/03/23 and by the Interdisciplinary Center of Clinical Research of the Medical Faculty Jena.

## Declaration of competing interest

The authors declare that they have no known competing financial interests or personal relationships that could have appeared to influence the work reported in this paper.

## Data Availability

The data underlying this article are available in the article and in its online supplementary material.

## References

[1] Murtha L.A., Schuliga M.J., Mabotuwana N.S., Hardy S.A., Waters D.W., Burgess J.K. (2017). The processes and mechanisms of cardiac and pulmonary fibrosis. Front. Physiol..

[2] Disertori M., Masè M., Ravelli F. (2017). Myocardial fibrosis predicts ventricular tachyarrhythmias. Trends Cardiovasc. Med..

[3] Jellis C., Martin J., Narula J., Marwick T.H. (2010). Assessment of nonischemic myocardial fibrosis. J. Am. Coll. Cardiol..

[4] Dweck M.R., Kwiecinski J. (2019). Emerging sex differences in aortic stenosis. JACC Cardiovasc. Imaging.

[5] Bhuva A.N. (2020). Sex differences in myocardial fibrosis in aortic stenosis. J. Am. Coll. Cardiol..

[6] Kwak S. (2025). Gender-specific patterns of myocardial remodeling in aortic stenosis. Circulation.

[7] d’Arcy J.L., Coffey S., Loudon M.A., Kennedy A., Pearson-Stuttard J., Birks J. (2016). Large-scale community echocardiographic screening reveals a major burden of undiagnosed valvular heart disease in older people: the OxVALVE Population Cohort Study. Eur. Heart J..

[8] Osnabrugge R.L., Mylotte D., Head S.J., Van Mieghem N.M., Nkomo V.T., LeReun C.M. (2013). Aortic stenosis in the elderly: disease prevalence and number of candidates for transcatheter aortic valve replacement: a meta-analysis and modeling study. J. Am. Coll. Cardiol..

[9] Lee J.M., Park S.J., Lee S.P., Park E., Chang S.A., Kim H.K. (2015). Gender difference in ventricular response to aortic stenosis: insight from cardiovascular magnetic resonance. PLoS One.

[10] Treibel T.A., Kozor R., Fontana M., Torlasco C., Reant P., Badiani S. (2018). Sex dimorphism in the myocardial response to aortic stenosis. JACC Cardiovasc. Imaging.

[11] Page M.J., McKenzie J.E., Bossuyt P.M., Boutron I., Hoffmann T.C., Mulrow C.D. (2021). The PRISMA 2020 statement: an updated guideline for reporting systematic reviews. BMJ.

[12] Page M.J., Moher D., Bossuyt P.M., Boutron I., Hoffmann T.C., Mulrow C.D. (2021). PRISMA 2020 explanation and elaboration: updated guidance and exemplars for reporting systematic reviews. BMJ.

[13] G. Wells, The Newcastle-Ottawa Scale (NOS) for assessing the quality of nonrandomised studies in meta-analysis. Ottawa: Ottawa Hospital Research Institute; 2004. Available at: http://www.ohri.ca/programs/clinical_epidemiology/oxford.asp.

[14] Guyatt G.H., Oxman A.D., Vist G.E., Kunz R., Falck-Ytter Y., Alonso-Coello P. (2008). GRADE: an emerging consensus on rating quality of evidence and strength of recommendations. BMJ.

[15] Singh A., Al Musa T., Treibel T.A., Vassiliou V.S., Captur G., Chin C. (2019). Sex differences in left ventricular remodelling, myocardial fibrosis and mortality after aortic valve replacement. Heart.

[16] Dobson L.E., Fairbairn T.A., Musa T.A., Uddin A., Mundie C.A., Swoboda P.P. (2016). Sex-related differences in left ventricular remodeling in severe aortic stenosis and reverse remodeling after aortic valve replacement: a cardiovascular magnetic resonance study. Am. Heart J..

[17] Tastet L., Kwiecinski J., Pibarot P., Capoulade R., Everett R.J., Newby D.E. (2020). Sex-related differences in the extent of myocardial fibrosis in patients with aortic valve stenosis. JACC Cardiovasc. Imaging.

[18] Dahou A., Awasthi V., Bkhache M., Djellal M., Yang X., Wang H. (2024). Sex-related differences in the pathophysiology, cardiac imaging, and clinical outcomes of aortic stenosis: a narrative review. J. Clin. Med..

[19] Petrov G., Regitz-Zagrosek V., Lehmkuhl E., Krabatsch T., Dunkel A., Dandel M. (2010). Regression of myocardial hypertrophy after aortic valve replacement: faster in women?. Circulation.

[20] Kararigas G., Dworatzek E., Petrov G., Summer H., Schulze T.M., Baczko I. (2014). Sex-dependent regulation of fibrosis and inflammation in human left ventricular remodelling under pressure overload. Eur. J. Heart Fail..

[21] Novella S., Gerdts E., Kararigas G. (2025). Divergent mechanisms of cardiovascular remodeling between men and women. Am. J. Physiol. Heart Circ. Physiol..

[22] Faggiano A., Gherbesi E., Carugo S., Brusamolino M., Cozac D.A., Cozza E. (2025). Prognostic value of myocardial computed tomography–derived extracellular volume in severe aortic stenosis requiring aortic valve replacement: a systematic review and meta-analysis. Eur. Heart J. Cardiovasc. Imaging.

