# Supplementary material for: Sex-related differences in myocardial fibrosis among patients with aortic stenosis: A systematic review and meta-analysis
Source: Int J Cardiol Heart Vasc. 2025 Sep 30;61:101814. doi: 10.1016/j.ijcha.2025.101814 (PMC12513023; doi:10.1016/j.ijcha.2025.101814)

**Supplementary material**

**Supplementary Table 1.** Complete search strategy

**Supplementary Table 2**. GRADE overview

**Supplementary Figure 1.** Assessment of risk of bias using the Newcastle Ottawa Scale

**Supplementary Figure 2**. Funnel Plot for Publication Bias Assessment. (A) LGE%. (B) infarct-related LGE. (C) non-infarct LGE

**Supplementary Figure 3**. Leave-one-out sensitivity analysis for LGE%

**Supplementary Figure 4**. Leave-one-out sensitivity analysis for ECV

**Supplementary Figure 5**. Leave-one-out sensitivity analysis for Septal E/e’

**Supplementary Figure 6**. Subgroup Analysis by NOS Score for LGE%

**Supplementary Figure 7**. Subgroup Analysis by NOS Score for Infarct-related LGE

**Supplementary Figure 8**. Subgroup Analysis by NOS Score for Non-infarct-related LGE

**Supplementary Figure 9**. Subgroup Analysis by NOS Score for ECV

**Supplementary Figure 10**. Subgroup Analysis by NOS Score for Septal E/e’

**Supplementary Table 1.**

("myocardially"[All Fields] OR "myocardium"[MeSH Terms] OR "myocardium"[All Fields] OR "myocardial"[All Fields]) AND ("fibrosi"[All Fields] OR "fibrosing"[All Fields] OR "fibrosis"[MeSH Terms] OR "fibrosis"[All Fields] OR "fibrose"[All Fields] OR "fibroses"[All Fields]) AND ("aortic valve stenosis"[MeSH Terms] OR ("aortic"[All Fields] AND "valve"[All Fields] AND "stenosis"[All Fields]) OR "aortic valve stenosis"[All Fields] OR ("aortic"[All Fields] AND "stenosis"[All Fields]) OR "aortic stenosis"[All Fields]) AND ("gender identity"[MeSH Terms] OR ("gender"[All Fields] AND "identity"[All Fields]) OR "gender identity"[All Fields] OR "gendered"[All Fields] OR "gender s"[All Fields] OR "gendering"[All Fields] OR "genderized"[All Fields] OR "genders"[All Fields] OR "sex"[MeSH Terms] OR "sex"[All Fields] OR "gender"[All Fields] OR ("sex characteristics"[MeSH Terms] OR ("sex"[All Fields] AND "characteristics"[All Fields]) OR "sex characteristics"[All Fields] OR ("sex"[All Fields] AND "differences"[All Fields]) OR "sex differences"[All Fields]))

**Translations**

**myocardial:** "myocardially"[All Fields] OR "myocardium"[MeSH Terms] OR "myocardium"[All Fields] OR "myocardial"[All Fields]

**fibrosis:** "fibrosi"[All Fields] OR "fibrosing"[All Fields] OR "fibrosis"[MeSH Terms] OR "fibrosis"[All Fields] OR "fibrose"[All Fields] OR "fibroses"[All Fields]

**aortic stenosis:** "aortic valve stenosis"[MeSH Terms] OR ("aortic"[All Fields] AND "valve"[All Fields] AND "stenosis"[All Fields]) OR "aortic valve stenosis"[All Fields] OR ("aortic"[All Fields] AND "stenosis"[All Fields]) OR "aortic stenosis"[All Fields]

**gender:** "gender identity"[MeSH Terms] OR ("gender"[All Fields] AND "identity"[All Fields]) OR "gender identity"[All Fields] OR "gendered"[All Fields] OR "gender's"[All Fields] OR "gendering"[All Fields] OR "genderized"[All Fields] OR "genders"[All Fields] OR "sex"[MeSH Terms] OR "sex"[All Fields] OR "gender"[All Fields]

**sex differences:** "sex characteristics"[MeSH Terms] OR ("sex"[All Fields] AND "characteristics"[All Fields]) OR "sex characteristics"[All Fields] OR ("sex"[All Fields] AND "differences"[All Fields]) OR "sex differences"[All Fields]

**Supplementary Table 2**.

1. **Risk of Bias:** The studies assessed were observational and received a moderate quality rating according to the Newcastle–Ottawa Scale (NOS). Some studies had unclear blinding or potential for selective reporting, particularly in imaging-based outcomes.
2. **Inconsistency**: Moderate to substantial heterogeneity observed in several outcomes (e.g., I² > 50%), which may reflect clinical or methodological differences between studies.
3. **Imprecision**: Some estimates had wide confidence intervals overlapping the null effect, reflecting statistical uncertainty due to small sample sizes or event rates.
4. **Indirectness**: Not downgraded; all included studies investigated directly relevant populations, interventions, and outcomes consistent with the research objective.
5. **Publication Bias**: Funnel plots for each outcome were visually symmetric, and no asymmetry was observed. We did not perform Egger's test because there were only about 10 studies for each outcome

| **Study** | **Selection (**★**/4)** | **Comparability (**★**/2)** | **Outcome (**★**/3)** | **Total Stars (**★**/9)** | **Quality Rating** |
| --- | --- | --- | --- | --- | --- |
| Bhuva et al., 2019 | ★★★★ | ★★ | ★★★ | **9/9** | High |
| Dobson et al., 2016 | ★★★★ | ★★ | ★★ | **8/9** | High |
| Kwak et al., 2025 | ★★★★ | ★★ | ★★★ | **9/9** | High |
| Lee et al., 2015 | ★★ | ★★ | ★ | **5/9** | Low to Moderate |
| Singh et al., 2019 | ★★★★ | ★★ | ★★★ | **9/9** | High |
| Treibel et al., 2018 | ★★★★ | ★★ | ★ | **7/9** | Moderate to High |
| Tastet et al., 2020 | ★★★ | ★★ | ★ | **6/9** | Moderate |

| Outcome | No. Participants (Studies) | Relative Effect (95% CI) | Mean / Risk in Females | Mean / Risk in Males | Difference | Certainty of Evidence (GRADE) | Interpretationon |
| --- | --- | --- | --- | --- | --- | --- | --- |
| LGE% of Myocardium | 1,979  (6 studies) | MD 0.17 (–0.45 to 0.79) | — | — | 0.17% more (–0.45 to 0.79) | ⨁◯◯◯ Very Low¹²³ | May result in little or no difference |
| Extracellular Volume (ECV) | 1,203  (4 studies) | MD –0.45 (–1.52 to 0.63) | — | — | 0.45% less (–1.52 to 0.63) | ⨁◯◯◯ Very Low¹²³ | May result in little or no difference |
| Infarct-related LGE | 1,612  (4 studies) | RR 1.61 (1.12 to 2.31) | 5.8% | 9.2% | 3.4% more (0.7 to 6.0) | ⨁⨁◯◯ Low²³ | May be associated with increased risk in males |
| Non-infarct-related LGE | 1,761  (4 studies) | RR 1.51 (1.31 to 1.74) | 12.2% | 18.4% | 6.2% more (4.0 to 8.5) | ⨁⨁◯◯ Low²³ | Probably more prevalent in males |
| E/e′ (Septal) | 1,207  (4 studies) | MD –1.87 (–3.16 to –0.58) | — | — | Lower in males (–1.87 units) | ⨁⨁◯◯ Low²³ | Likely lower in males |
| Publication Bias (Funnel Plot Assessment) | — | Asymmetry not evident | — | — | — | — | No significant small-study effects observed |


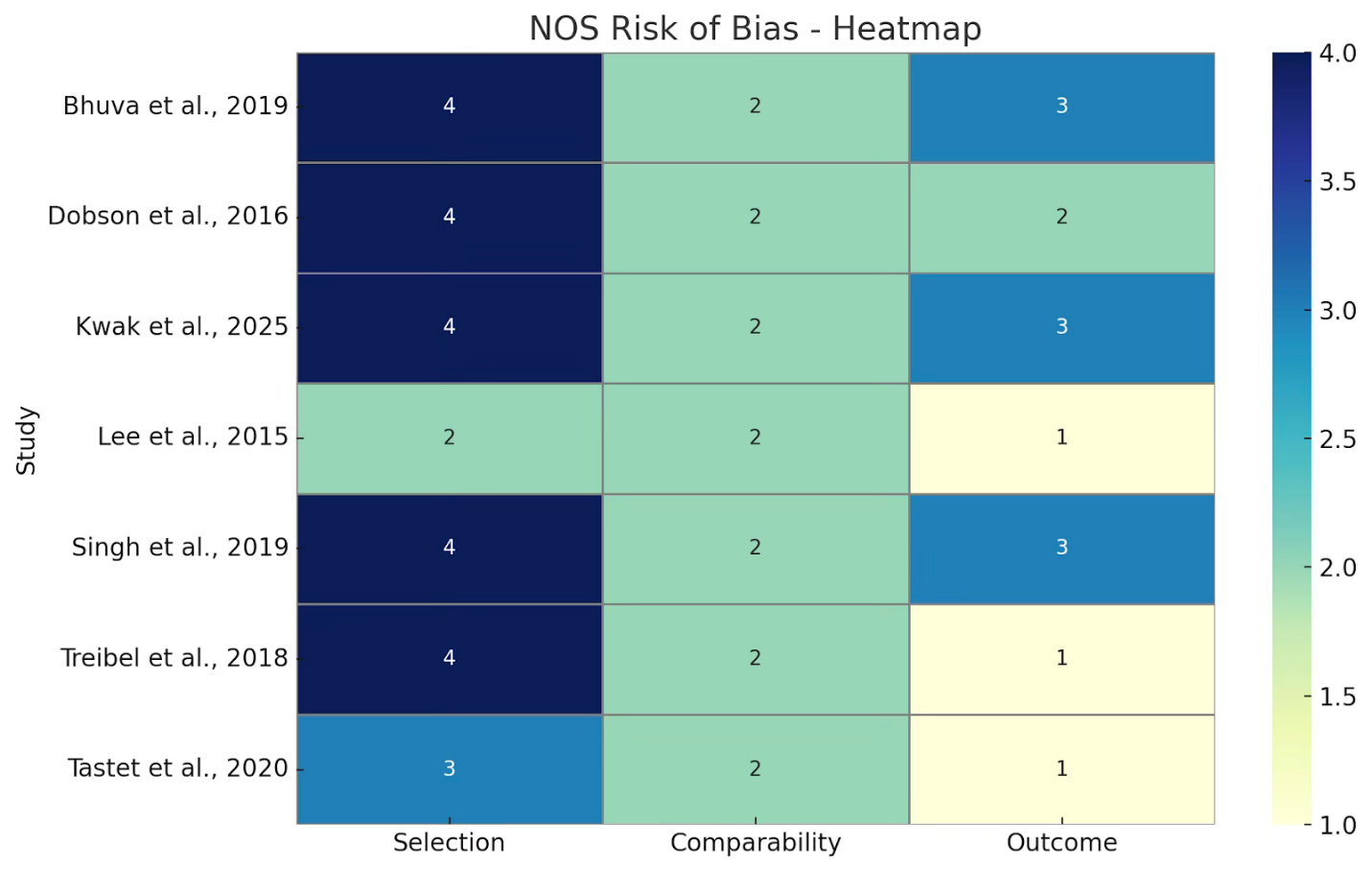
**Supplementary Figure 1.**

**Supplementary Figure 2.**

**A.**

**
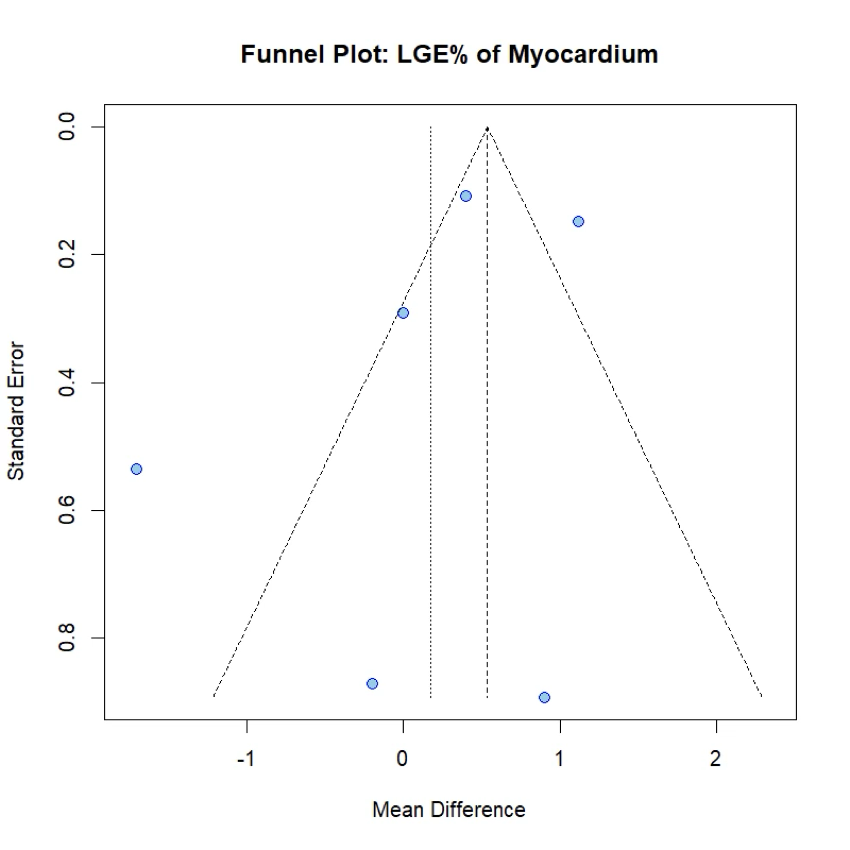
**

**B.**


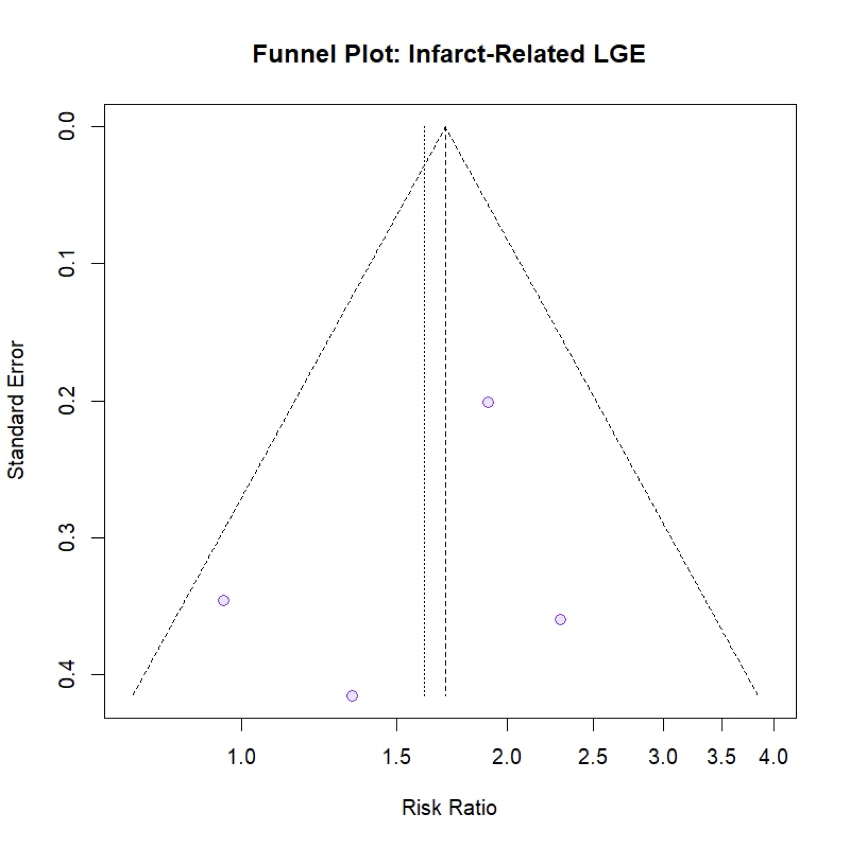


**C.**


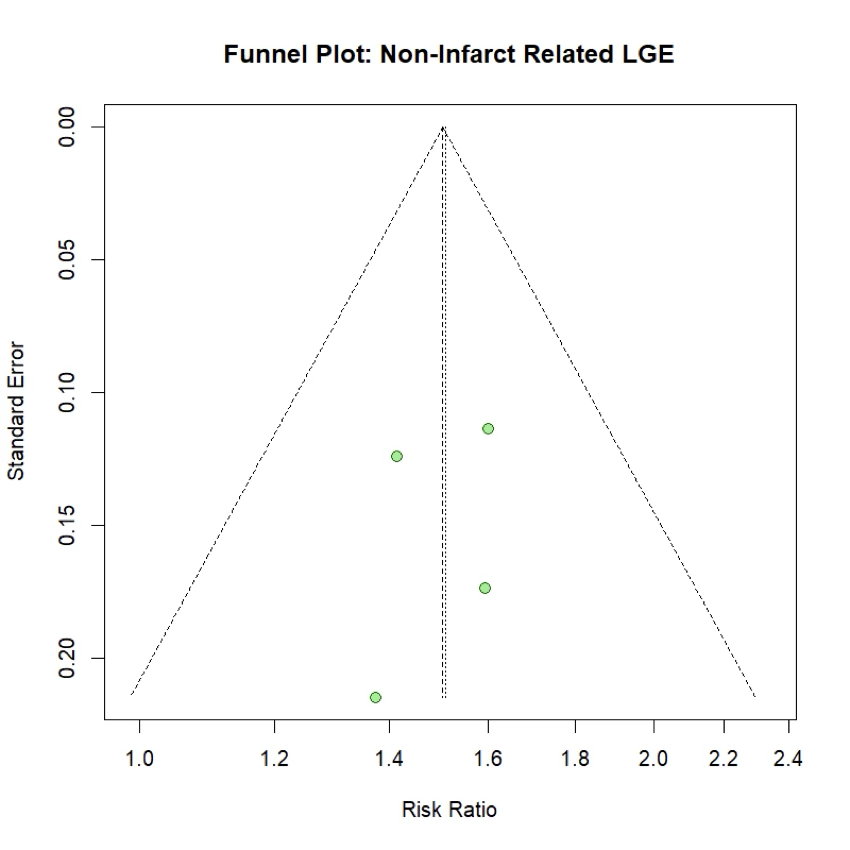


**
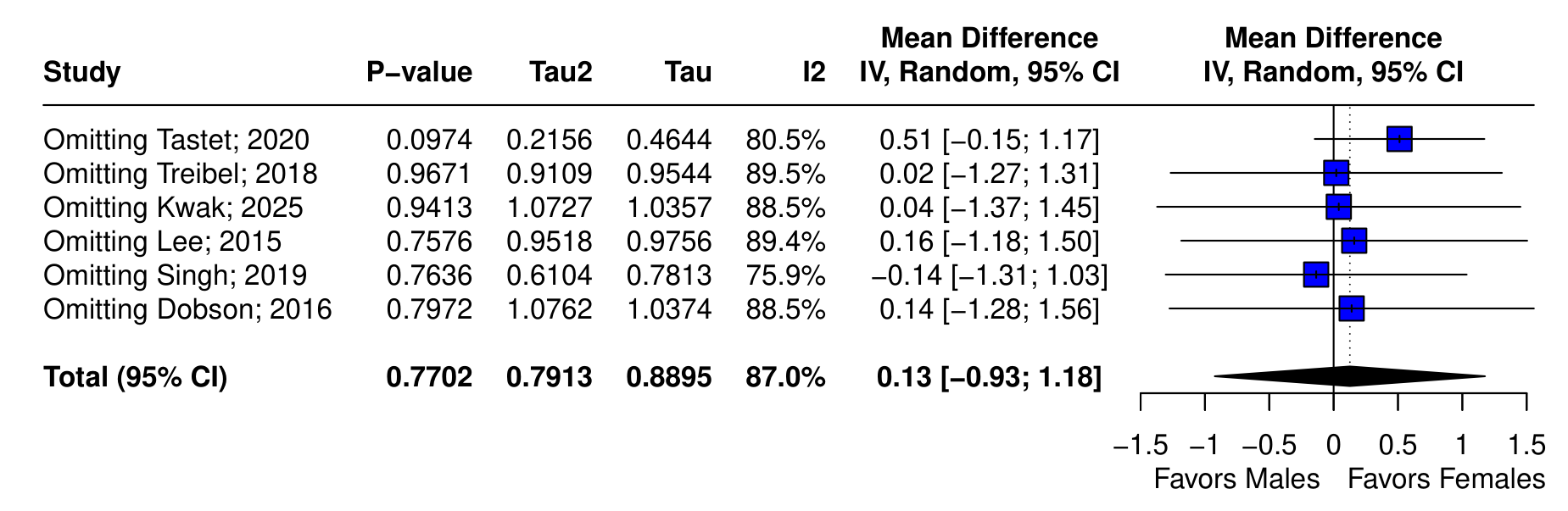
Supplementary Figure 3.**

**
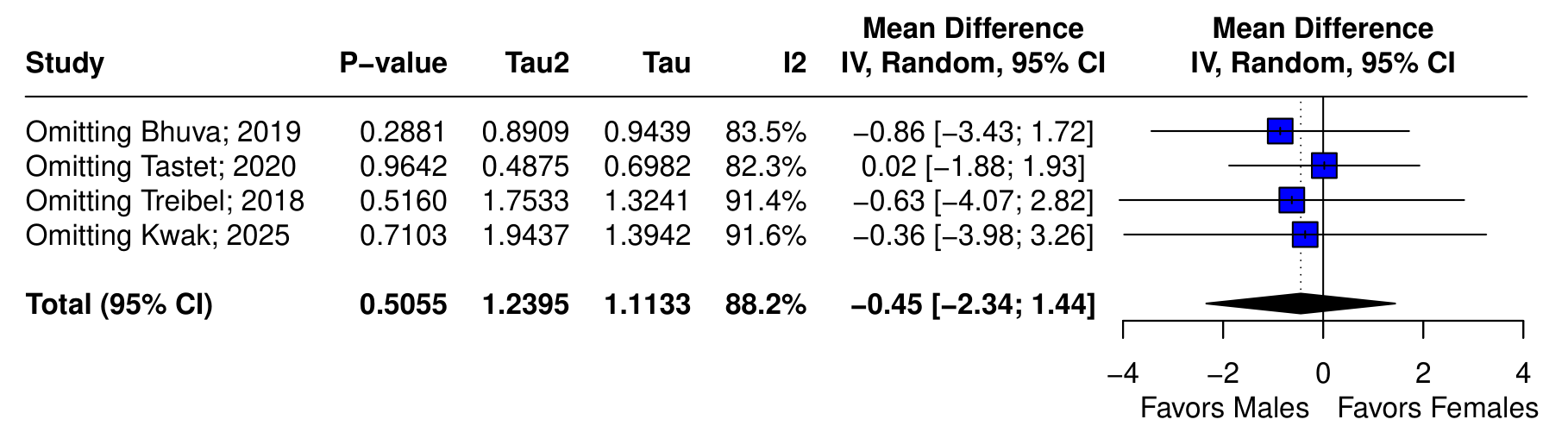
Supplementary Figure 4.**

**
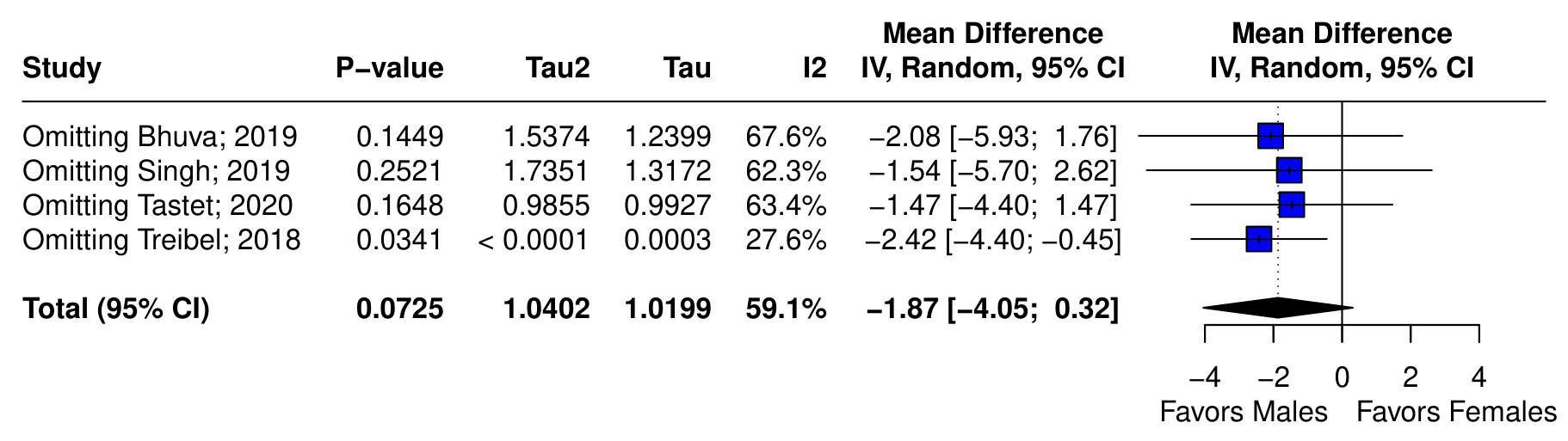
Supplementary Figure 5.**

**
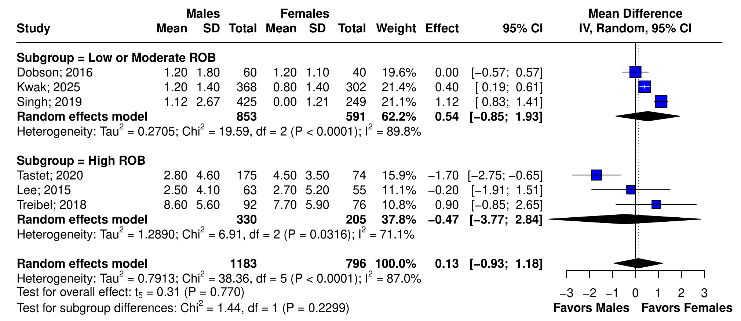
Supplementary Figure 6.**

**
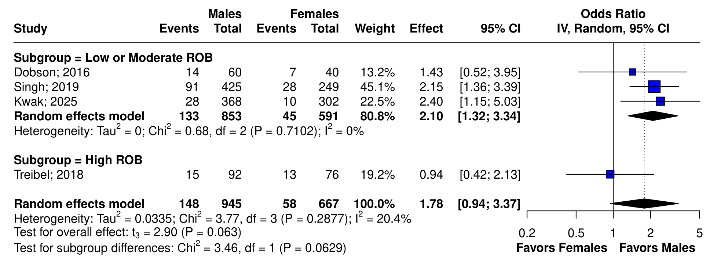
Supplementary Figure 7.**

**
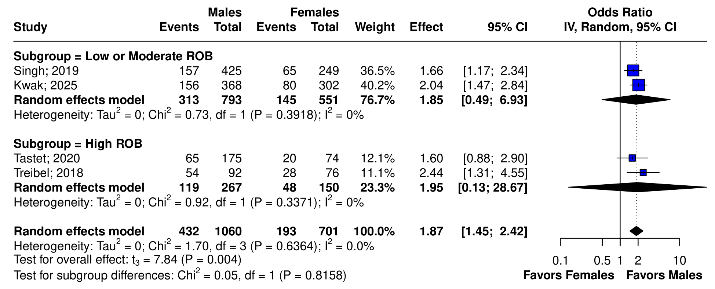
Supplementary Figure 8.**

**
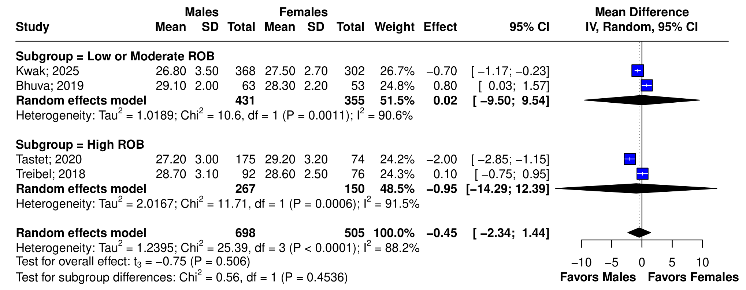
Supplementary Figure 9.**

**Supplementary Figure 10.**


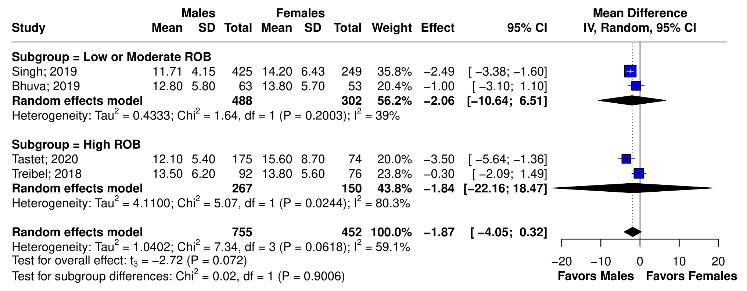

Supplement: Supplementary Data 1 [file mmc1.docx]
